# c-KIT positive Gastrointestinal Stromal Tumor presenting with acute bleeding in a patient with neurofibromatosis type 1: a case report

**DOI:** 10.1186/1477-7800-6-17

**Published:** 2009-10-23

**Authors:** Esam Aboutaleb, Manish Kothari, Osama Damrah, Roben Canelo

**Affiliations:** 1 Imperial College Healthcare Trust, Hammersmith Hospital, Department of Hepatopancreatic and Biliary Surgery, Du Cane Road, London, W12 0HS, UK

## Abstract

**Background:**

Gastrointestinal stromal tumours are rare (GIST). However, the incidence of GIST among neurofibromatosis type 1 (NF-1) patients is approximately 3.9-25%. GIST can present clinically in different ways such as abdominal pain, gastrointestinal bleeding and obstruction.

**Case report:**

We present 51 year female patient admitted with Background of neurofibromatosis type 1 admitted with melena. OGD has been done and showed duodenitis with large volume fresh blood in distal duodenum but no obvious bleeding point. Exploratory laparotomy revealed smooth nodular masses on the serosal surface of jejunum. Small bowel resection and side-to-side anastomosis were performed. Histopathoogical examination revealed small bowel gasrointestinal stromal tumour with low risk malignant potential.

**Conclusion:**

The incidence of GIST among neurofibromatosis type 1 (NF-1) patients is not uncommon and we should pay attention to gastrointestinal manifestation in such patients.

## Introduction

Neurofibromatosis is an autosomal dominant disorder that affects all 3 germinal layers; thereby affecting any organ system. The National Institutes of Health (NIH) Consensus Development Conference has defined 2 distinct types: the commoner of the two, neurofibromatosis type 1 (NF1) affecting 85% of patients; and neurofibromatosis type 2 (NF2) [1]. NF1diagognsis made if 2 or more of following criteria found: Six or more café au lait macules larger than 5 mm, two or more neurofibromas, multiple freckles, A distinctive osseous lesion, optic glioma, two or more iris hamartomas, a first-degree relative with NF1. Rarely, the neurofibromatosis may become cancerous (3-5%). Gastrointestinal stromal tumours (GIST) are generally rare however; the incidence of GIST among NF-1 patients is approximately 5-25% [2]. GISTs are usually located in the stomach and small intestine and can present in a variety of different ways ranging from vague symptoms to major G.I. bleeding. The first line treatment is surgical resection for operable GIST and 5-year survival ranges from 21% to 88% depending on risk grading and completeness of surgical resection [3,4]. The second line of treatment is Imatinib mesylate, a tyrosine kinase inhibitor, which provides an option for treating high risk GISTs.

We report the case of a 51 year female patient with NF-1 who presented with lower GI bleeding caused by a jejunal GIST.

## Case report

A 51 year old lady with NF-1 as she has multiple neurofibromas and seven café au lait macules larger than 5 mm. Presented to A&E with a 24 hour history of malena. She was on warfarin for aortic root and valve replacement surgery she had in the previous year. Although she remained haemodynamically stable, her haemoglobin levels gradually dropped 4 units over the next 36 hours. Along with correction of her clotting function, investigations were initiated that included an upper GI endoscopy which revealed blood in the second part of duodenum. Due to a rapid deterioration in her haemodynamic status colonoscopy was cancelled and a mesenteric angiogram was done that suggested bleeding from the region between the 4^th ^part of duodenum and upper jejunum from intestinal mass. She was subjected to emergency laparotomy. At surgery, smooth nodular masses were found on the serosal surface of the first 50 cm of jejunum; these were 3-4 cm and projected into the lumen having eroded the mucosal surface. Resection of the affected segment and end to end anastomosis was performed. The rest of the bowel appeared free of any masses and after concluding surgery the patient was transferred to ITU postoperatively. She made an uncomplicated recovery and was discharged 7 days after operation. A subsequent CT scan did not reveal any other masses.

**Figure 1:**
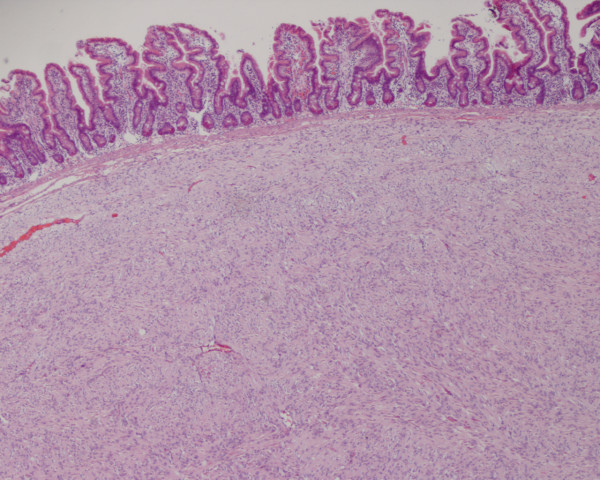
**Gastro-intestinal stromal tumour (GIST) in small bowel, arising from the submucosa (haematoxylin and eosin; original magnification × 40)**.

Histopathology revealed an 8 cm length of jejunum involved with tumour nodules, smallest 3.4 cm and largest 4.4 cm, composed of spindle and polygonal cells. The tumour involved the full thickness of the bowel wall and there was no spread of disease to lymph nodes. Immunohistochemistry demonstrated positive staining for CD117 (cKit) (Figure 1), and negative staining for broad range cytokeratins; MNF 116, CD34, desmin, S-100 and chromogranin. On the basis of mitotic count (2/50 HPF) and tumour size it was classified as a GIST of intermediate malignant potential. Proliferation index detected by Ki67 was very low (less than 1-2%).

## Discussion

Abdominal involvement in NF-1 has been described before. This includes GISTs [5], pheochromocytomas [6] and ampullary tumours [7]. 78% of sporadic GISTs show c-KIT mutations [8]. However, this typical mutation is rarely seen in NF-1 associated GISTs [5,9] leading to authors proposing an alternative pathogenesis of GISTs in NF-1 patients [10]. This has potential implications for Imatinib therapy when required as this is likely to be ineffective in c-KIT negative ones as in NF-1 [11].

Figure 1- Gastro-intestinal stromal tumour (GIST) in small bowel, arising from the submucosa (haematoxylin and eosin; original magnification × 40)

**Figure 2:**
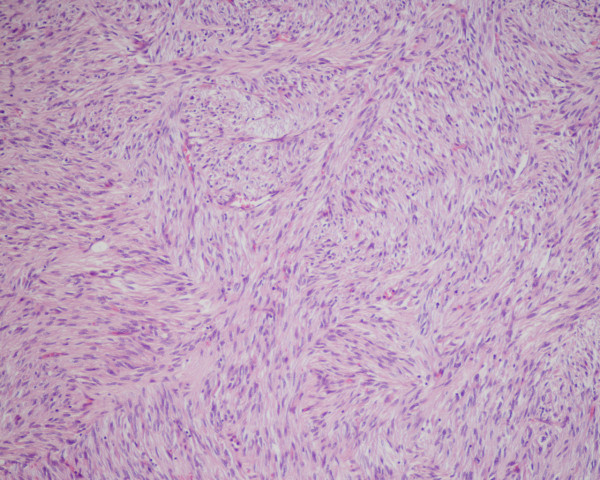
**The tumour is composed of spindle cells with plump nuclei and abundant eosinophilic cytoplasm with fibrovascular stroma (haematoxylin and eosin; original magnification × 100)**.

Figure 2- The tumour is composed of spindle cells with plump nuclei and abundant eosinophilic cytoplasm with fibrovascular stroma (haematoxylin and eosin; original magnification × 100)

**Figure 3:**
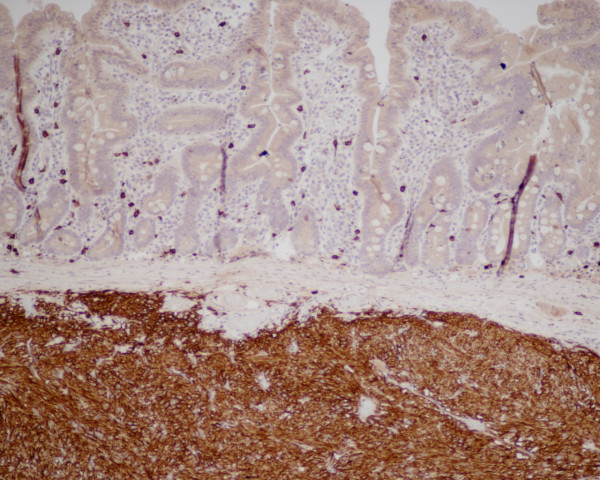
**Immunohistochemical staining for CD117 (A) and S100 protein (B)**. The tumour cells show strong diffuse membranous and cytoplasmic expression for CD117 and negative expression for S100 protein.

**Figure 4:**
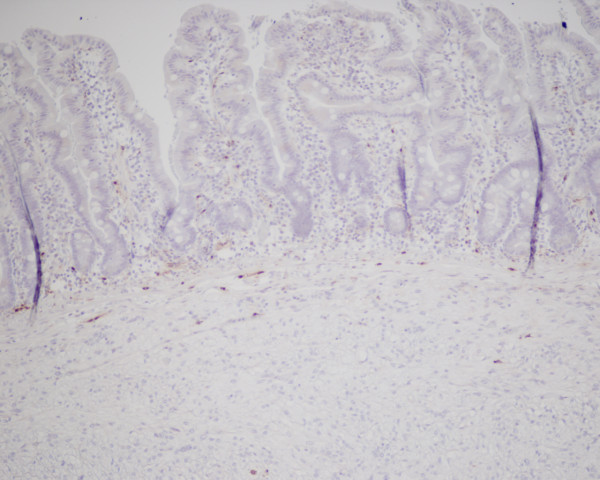
**Immunohistochemical staining for CD117 (A) and S100 protein (B)**. The tumour cells show strong diffuse membranous and cytoplasmic expression for CD117 and negative expression for S100 protein.

Figure 3 and Figure 4. Immunohistochemical staining for CD117 (A) and S100 protein (B). The tumour cells show strong diffuse membranous and cytoplasmic expression for CD117 and negative expression for S100 protein

Our patient possessed the known c-KIT mutation that does not appear typical of NF-1 associated GISTs from review of literature. It may be that our patient developed a GIST by co-incidence rather than as an association with NF-1. However the finding of a low malignant potential GIST in our patient based on mitotic count is consistent with other NF-1 associated GISTs reported, along with other features like young age and female preponderance [5].

## Consent

Written informed consent was obtained from the patient for publication of this case report and accompanying images. A copy of the written consent is available for review by the Editor-in-Chief of this journal.

## Competing interests

The authors declare that they have no competing interests.

## Authors' contributions

EA participated in the sequence alignment and drafted the manuscript. MK participated in the sequence alignment. OD participated in the design of the study. RC conceived of the study, and participated in its design and coordination. All authors have read and approved the manuscript.
